# Functional morphology of immature mating in a widow spider

**DOI:** 10.1186/s12983-021-00404-1

**Published:** 2021-04-26

**Authors:** Lenka Sentenská, Aileen Neumann, Yael Lubin, Gabriele Uhl

**Affiliations:** 1 Department of General and Systematic Zoology, University of Greifswald, Loitzer Strasse 26, 17489 Greifswald, Germany; 2 Department of Biological Sciences, University of Toronto Scarborough, Scarborough, Ontario Canada; 3 Mitrani Department of Desert Ecology, Blaustein Institutes for Desert Research, Ben-Gurion University of the Negev, Sede Boqer Campus, Israel

**Keywords:** Araneae, Copulation, Immature mating, Alternative mating tactic, Histology, Spermathecae, Mating plug

## Abstract

**Background:**

Mating generally occurs after individuals reach adulthood. In many arthropods including spiders, the adult stage is marked by a final moult after which the genitalia are fully developed and functional. In several widow spider species (genus *Latrodectus*), however, immature females may mate a few days before they moult to adulthood, i.e. in their late-subadult stage. While the “adult” mating typically results in cannibalism, males survive the “immature” mating. During both “immature” and “adult” matings, males leave parts of their paired copulatory organs within female genitalia, which may act as mating plugs. To study potential costs and benefits of the two mating tactics, we investigated female genital morphology of the brown widow spider, *L. geometricus*. Light microscopy, histology and micro-computed tomography of early-subadult, late-subadult and adult females were conducted to determine the overall pattern of genital maturation. We compared genitalia of mated late-subadult and adult females to reveal potential differences in the genitalic details that might indicate differential success in sperm transfer and different environments for sperm storage and sperm competition.

**Results:**

We found that the paired sperm storage organs (spermathecae) and copulatory ducts are developed already in late-subadult females and host sperm after immature mating*.* However, the thickness of the spermathecal cuticle and the staining of the secretions inside differ significantly between the late-subadult and adult females. In late-subadult females mating plugs were found with higher probability in both spermathecae compared to adult females.

**Conclusions:**

Sperm transfer in matings with late-subadult females follows the same route as in matings with adult females. The observed differences in the secretions inside the spermathecae of adult and late-subadult females likely reflect different storage conditions for the transferred sperm which may lead to a disadvantage under sperm competition if the subadult female later re-mates with another male. However, since males mating with late-subadult females typically transfer sperm to both spermathecae they might benefit from numerical sperm competition as well as from monopolizing access to the female sperm storage organs. The assessment of re-mating probability and relative paternity will clarify the costs and benefits of the two mating tactics in light of these findings.

**Supplementary Information:**

The online version contains supplementary material available at 10.1186/s12983-021-00404-1.

## Background

Various mechanisms that reduce or prevent sperm competition have evolved in males [1, 2]. Apart from the common preference for virgin females, males reduce the re-mating probability of their mates by mate guarding, manipulating female receptivity or attractiveness to subsequent males, or by applying mechanical barriers such as mating plugs that hinder access to ova of their mates by other males. Some of these strategies prevent sperm competition but at the same time limit the male’s ability to mate again [3].

Males of two widow spider species, *Latrodectus hasselti* and *L. geometricus*, engage in an extreme form of paternity insurance through self-sacrifice [4, 5]. After inserting one of their paired copulatory organs (pedipalps), they somersault into the female’s fangs. Females attack and cannibalize males only if they adopt this position. Cannibalized males of *L. hasselti* benefit from higher relative paternity compared to their non-cannibalized competitors through longer copulations and decreased re-mating probability of the female, but are limited to mating with a single female [4].

Males of these two species break off parts of their copulatory organs inside the female genitalia [5, 6]. This type of plugging is common also in other widow spiders that lack self-sacrifice behaviour [7], as well as in several other spider species [8]. Widow spider males break off the tip of the embolus, i.e. the intromittent structure of their copulatory organ. When these embolus tips, sometimes called apical sclerites, are placed at the entrance to the female sperm storage sites (spermathecae) they may block insemination by rivals and serve as mating plugs. When the tips end up in the copulatory duct or inside the spermatheca they likely fail to prevent re-mating of the female [7]. The loss of the embolus tip does not prevent male widow spiders from inseminating subsequent females [7] in contrast to the genitalic mating plugs of many other spiders (e.g. [9]).

Mating behaviour in some widow spiders can be even more versatile. Recently a unique, cannibalism-free mating tactic was described in two species that exhibit the self-sacrifical behaviour (the Australian redback, *L. hasselti* and the brown widow, *L. geometricus*) [10, 11] and in one species lacking self-sacrifice (the western widow, *L. hesperus*) [12]. In these species, mating was observed with females at a late-subadult stage, during a narrow window of a few (2 to 6) days before the female undergoes the final moult to adulthood. Accordingly, the tactic was termed “immature mating” [10]. Males do not attempt to mate with early-stage-subadult females [13]. When mating with late-subadult females, males were observed to contact the region where the female copulatory structures are situated in the adult stage with their mouthparts. During “immature mating”, the cuticle in this region is ripped open [10, 14] which exposes the genital plate and allows the male to insert his copulatory organs. A dissection of the genital area of a late-subadult *L. hasselti* female suggested that the copulatory ducts and spermathecae are developed in this stage [10], however, it is not clear whether these genital structures are fully formed and can be used for sperm transfer and storage. Therefore, it is not clear whether a male uses the same copulatory route as with an adult female (i.e. insertion of the embolus into a copulatory duct that connects to a spermatheca) or whether he reaches the spermathecae or the eggs directly by so-called traumatic insemination similar to the spider *Harpactea sadistica* [15]. In *H. sadistica,* the male pierces female’s cuticle and transfers sperm that find their way to the ovaries through the hemolymph.

Immature matings lead to successful reproduction, since after moulting to adulthood, the *Latrodectus* females (*L. hasselti, L. hesperus, L. geometricus*) produce viable offspring [10–12, 14]. Mating with immature females has been observed in other spiders before and was termed “pseudocopulation” since sperm transfer was considered improbable [16, 17]. Indeed, such matings did not result in viable offspring in the spider *Anelosimus* cf. *studiosus* [18]. Therefore, the discovery of immature mating resulting in successful offspring production in the three widow spider species begs the question how it is achieved.

In all three widow spider species mentioned above, males are rarely cannibalized when mating with immature females. In the two self-sacrificial species (*L. hasselti, L. geometricus*), males that mate with subadult females do not somersault into the female’s mouthparts, as when mating with an adult female, thereby avoiding being cannibalized. Immature mating is further characterized by much-reduced courtship and a higher number of insertions of the male copulatory organs during copulation [10]. However, when given a choice, males of *L. geometricus* preferred to mate with adult females with whom they perform the somersault and are cannibalized [11, 13]. Since males recognize late-subadult females as potential mates, their preference for adult females suggests that immature mating entails costs. These costs could arise, for example, from the structural aspects of the developing genitalia, leading to disadvantages in transfer, storage and activation of sperm. A further potential cost of immature mating to the male is that these females must undergo a moult to adulthood, and by shedding the cuticle the stored sperm and/or the mating plugs might get lost. Spermathecae and copulatory ducts are cuticular structures that are generally shed with the rest of the exoskeleton during the moult, as seen in some spider taxa in which females can moult after reaching maturity [19, 20]. Immature-mated females of widow spiders, however, produced offspring [10–12, 14], demonstrating that at least some sperm is retained and used to fertilize the eggs.

In this study, we investigated the genital morphology of subadult and adult females of the brown widow spider, *L. geometricus*. We asked whether the genitalia of late-subadult females differ from those of adult females in anatomical structure and provide a different environment for sperm due to the ongoing process of genital maturation. We investigated whether the genital structures of the females are fully prepared for mating already at the end of the subadult stage, and where sperm are stored. We compared genitalia of early-subadult, late-subadult and adult virgin females to determine the overall pattern of genital maturation. We compared cuticle, epithelia and secretions of spermathecae of late-subadult with those of adult females. After mating, we further inspected late-subadult and adult stages for the presence of sperm, number of insertions and male genital plugs.

## Results

### External female genitalia

**Fig. 1:**
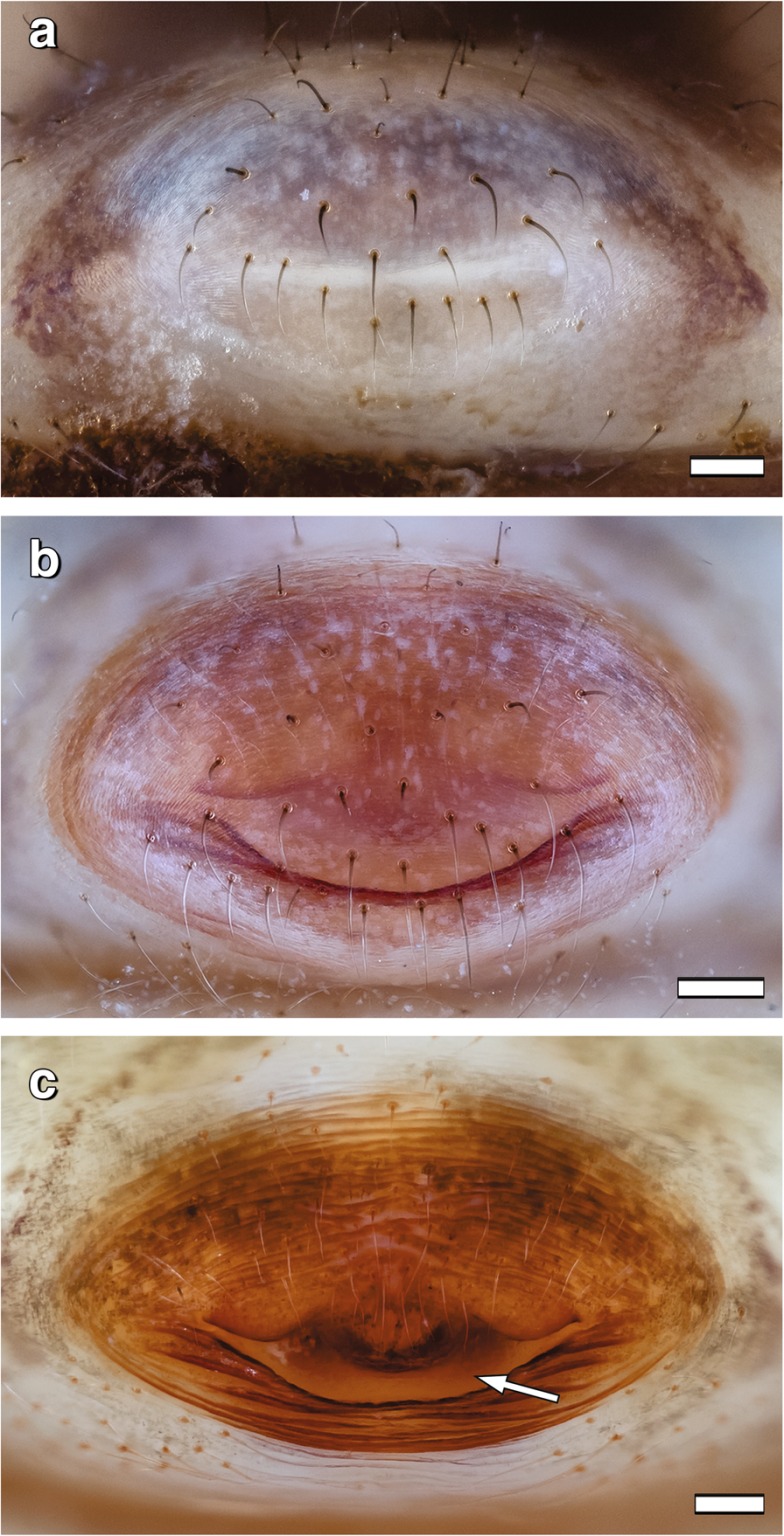
External genital region of virgin *L. geometricus* females in different developmental stages. Ventral view. Anterior is at the top, posterior is at the bottom. **a**: Early subadult female. **b**: Late subadult female. **c**: Adult female. Arrow indicates slit-like access to the atrium. Scale bars = 0.1 mm

**Fig. 2:**
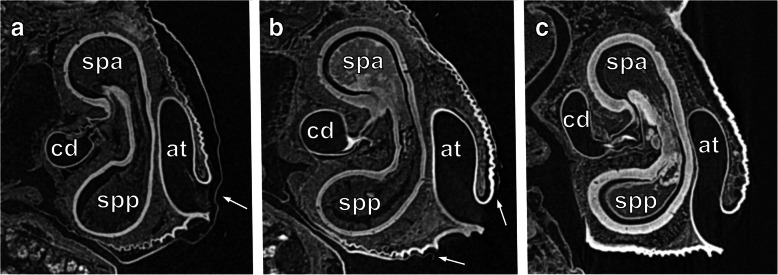
Virtual sagittal sections (Micro-CT Images) through the genital region of *L. geometricus* females in different developmental stages and of different mating status. Lateral view. Anterior is at the top, posterior is at the bottom. **a**: Virgin late-subadult female. Arrow indicates cuticle covering the genital atrium. **b**: Mated late-subadult female. Arrows indicate ripped open cuticle. **c**: Mated adult female. at, genital atrium; cd, copulatory duct; spa, anterior spermathecal lobe; spp., posterior spermathecal lobe

In early-subadult females (7 days after the moult to subadult stage), the genital area is slightly elevated forming a pale bulge (Fig. 1a). Approximately six days before the moult to the adult stage, this protuberance is darker (Fig. 1b). The dark coloured area indicates that the epigynal plate is formed and sclerotized underneath the exoskeleton already before the moult. In this stage, there is no access to the genitalia since the epigyne is still covered by body cuticle of the late-subadult females (Figs. 1b, 2a). During the final moult, the cuticle is shed, and the arched, dark brown, sclerotized epigynal plate with a slit-like access to the atrium and connected spermathecae is exposed (Figs. 1c, 2c). On each side of the atrium there is a broad entrance to a copulatory duct each leading to a bi-lobed spermatheca.

**Fig. 3:**
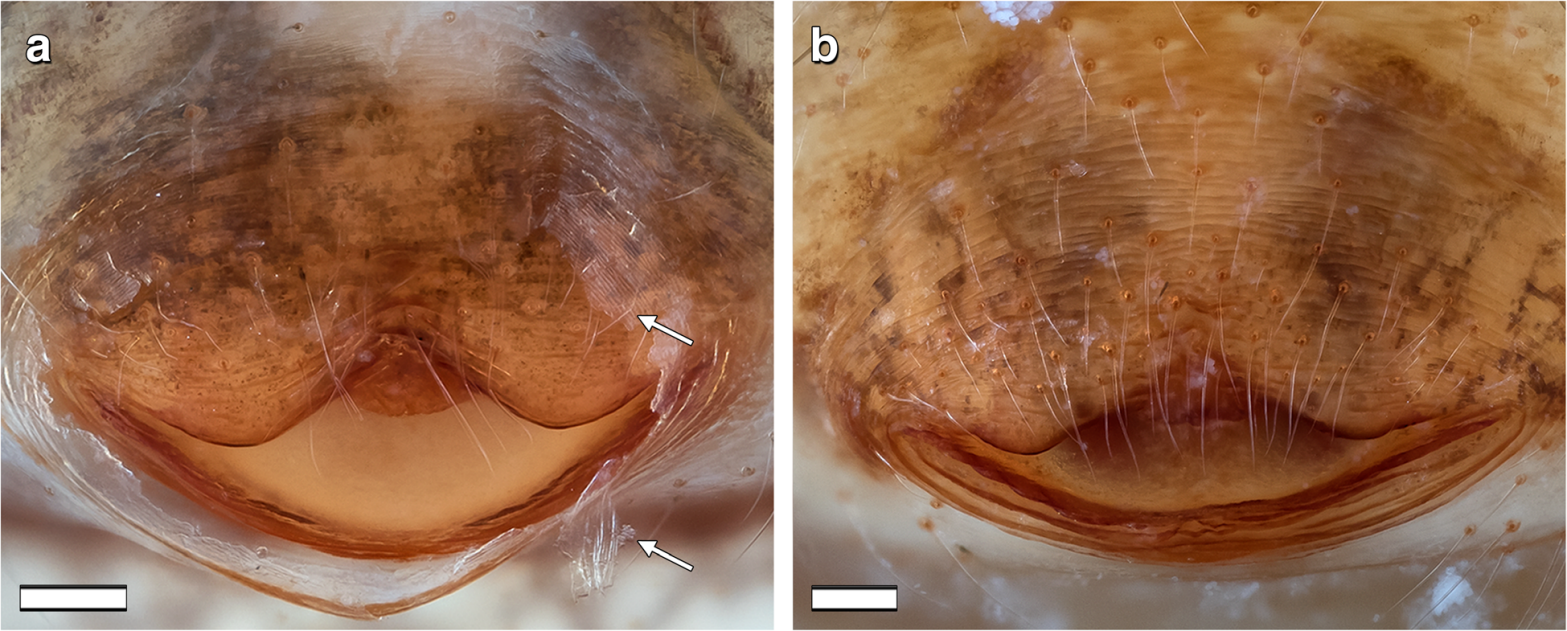
External genital region of mated *L. geometricus* females in different developmental stages. Ventral view. Anterior is at the top, posterior is at the bottom. **a**: Late-subadult female. Arrows indicate ripped open cuticle. **b**: Adult female. Scale bars = 0.1 mm


When matings occur with late-subadult females, the males disrupt the cuticle in the genital area by which the males gain access to the underlying epigyne and the connected spermathecae (Figs. 2 b, 3a; video S1). The disrupted cuticle is apparent in Fig. 2b.

**Additional file 3: Video S1**. Immature mating in *Latrodectus geometricus*. Male contacts female external genitalia (i.e. epigyne) with his chelicerae and copulatory organs; male bites through the cuticle covering the epigyne, exposing the atrium with the copulatory openings; male inserts one of his copulatory organs and transfers sperm; male withdraws the copulatory organ.

When females that mated as late-subadults (*N* = 15) eventually moult to the adult stage, their shed cuticle shows a rupture in the genital area. The underlying genital structures are not affected by the moulting process. When matings occur with adult females, their external genitalia do not differ before and after copulation (Fig. 1c versus 3B).

### Internal genitalia

#### General morphology

**Fig. 4:**
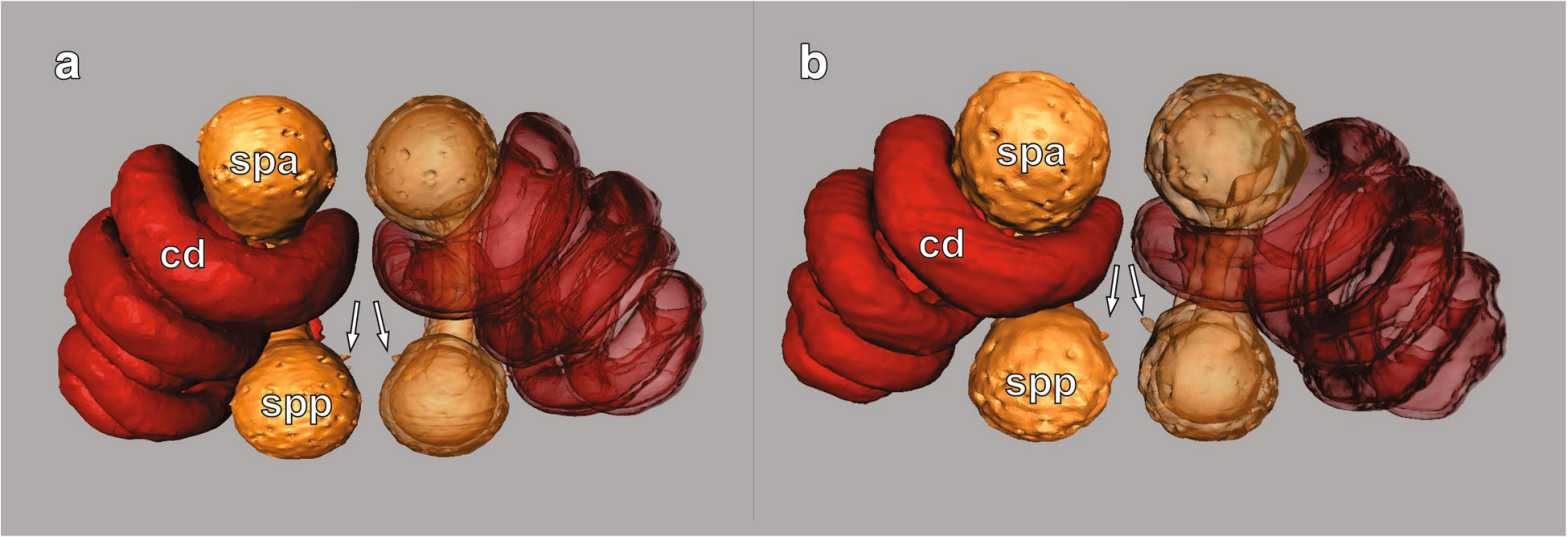
Sperm transfer and storage sites of mated late-subadult (**a**) and adult (**b**) *L. geometricus* females 3D-reconstructed using MicroCT analysis. Dorsal view. Surface view on the left side, transparent view on the right side. Anterior is at the top, posterior is at the bottom. Arrows indicate the fertilization ducts. cd, copulatory duct; spa, anterior spermathecal lobe; spp., posterior spermathecal lobe

**Fig. 5:**
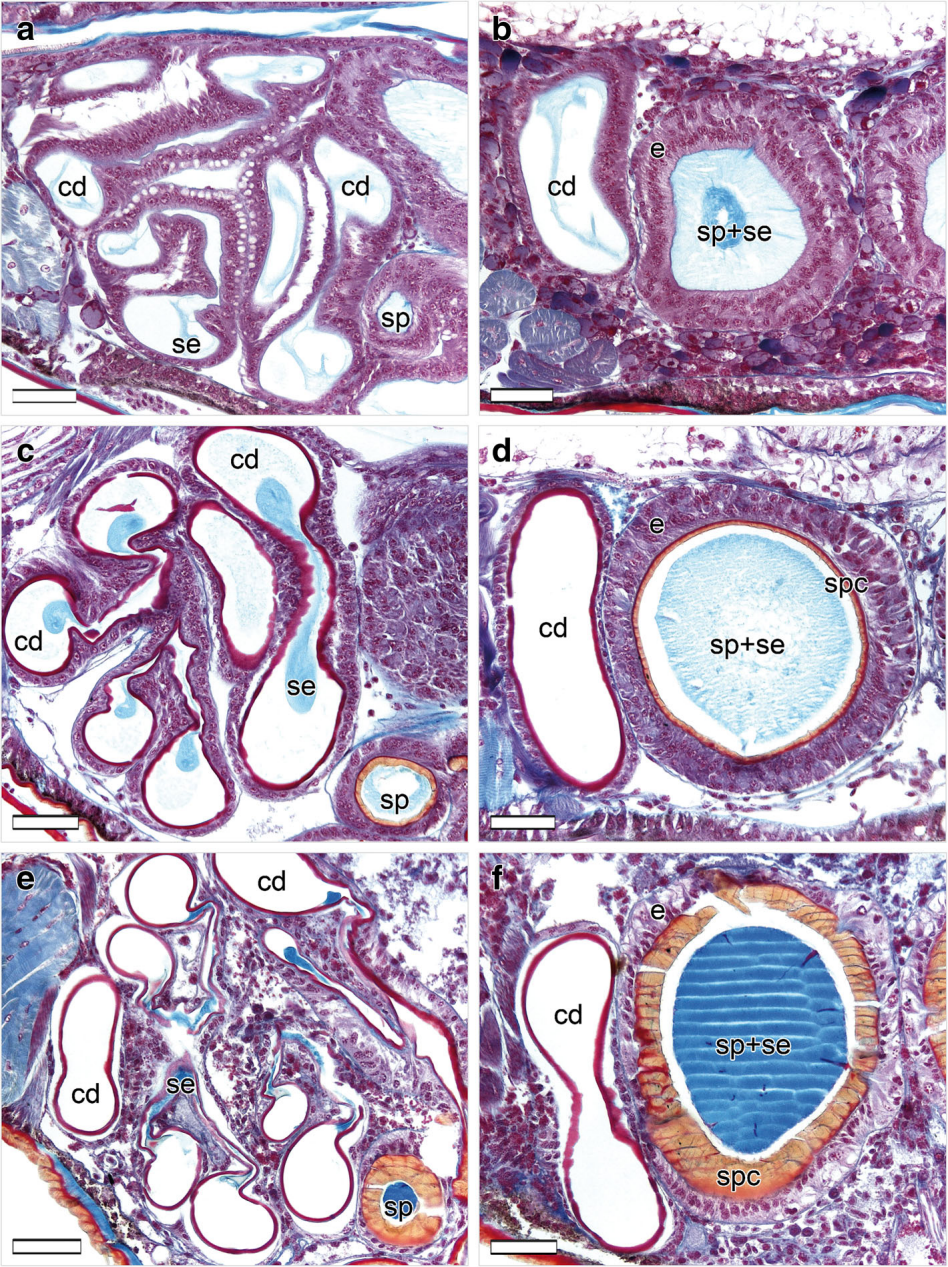
Sperm transfer and storage sites of virgin *L. geometricus* females in different developmental stages. Dorsal is at the top, ventral is at the bottom. **a**-**b**: Right copulatory duct and right spermatheca of an early-subadult female. Both structures are filled with a light blue secretion. **c**-**d**: Right copulatory duct and right spermatheca of a late-subadult female. Both structures are filled with a light blue secretion. **e**-**f**: Right copulatory duct and right spermatheca of an adult female. Both structures are filled with a bright blue secretion. Scale bars = 50 μm. cd, copulatory duct; e, epithelium; se, secretion; sp., spermatheca; spc, spermathecal cuticle

**Fig. 6:**
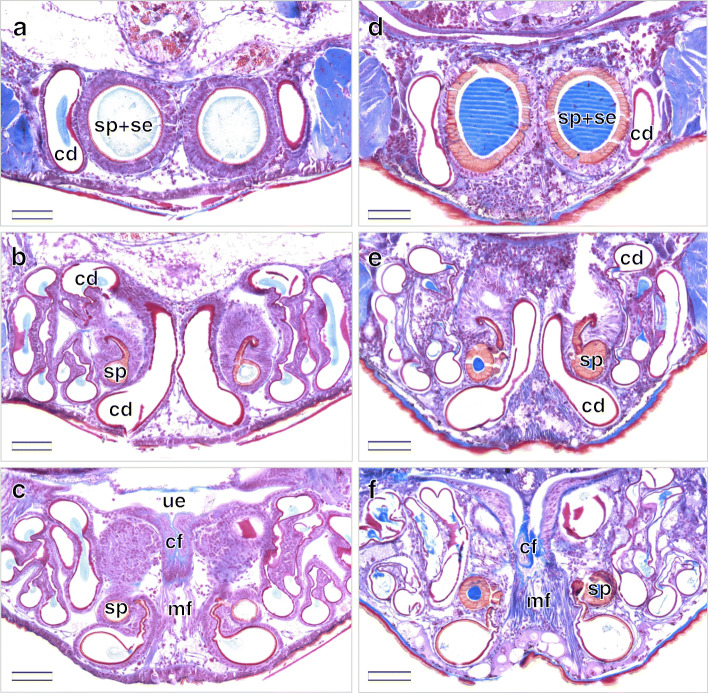
Serial cross sections of the genitalia of virgin *L. geometricus* females in different developmental stages starting from the anterior. Dorsal is at the top, ventral is at the bottom. **a**-**c**: Spermathecae and copulatory ducts of a late subadult female that are both filled with a light blue secretion. **d**-**f**: Spermathecae and copulatory ducts of an adult female that are both filled with a bright blue secretion. Scale bars = 100 μm. cd, copulatory duct; cf., common fertilization duct; mf, muscle fibers; se, secretion; sp., spermatheca; ue, uterus externus

**Fig. 7:**
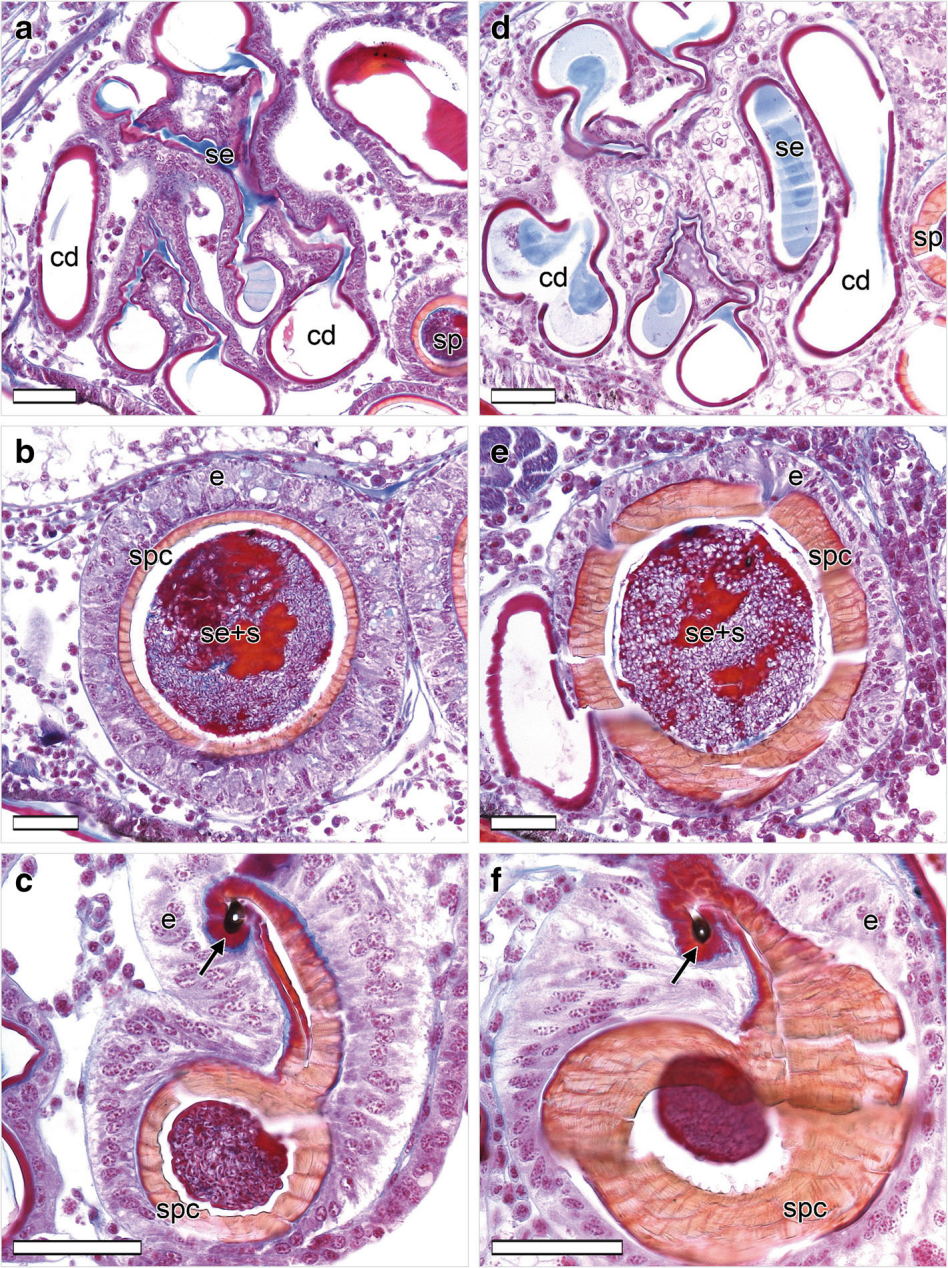
Details of genital structures of mated *L. geometricus* females in different developmental stages. Dorsal is at the top, ventral is at the bottom. **a**-**c**: Right copulatory duct and right spermatheca of a mated late subadult female. The spermatheca is completely filled with spermatozoa that are imbedded in a red-pink secretion. **c**: The male embolus tip (plug) is located inside the spermathecal entrance (arrow). **d**-**f**: Right copulatory duct and right spermatheca of a mated adult female. **e**: The spermatheca is filled with spermatozoa that are imbedded in a red-pink secretion. **f**: The male embolus tip (plug) is located inside the spermathecal entrance (arrow). Scale bars = 50 μm. cd, copulatory duct; e, epithelium; s, spermatozoa; se, secretion; sp., spermatheca; spc, spermathecal cuticle

**Fig. 8:**
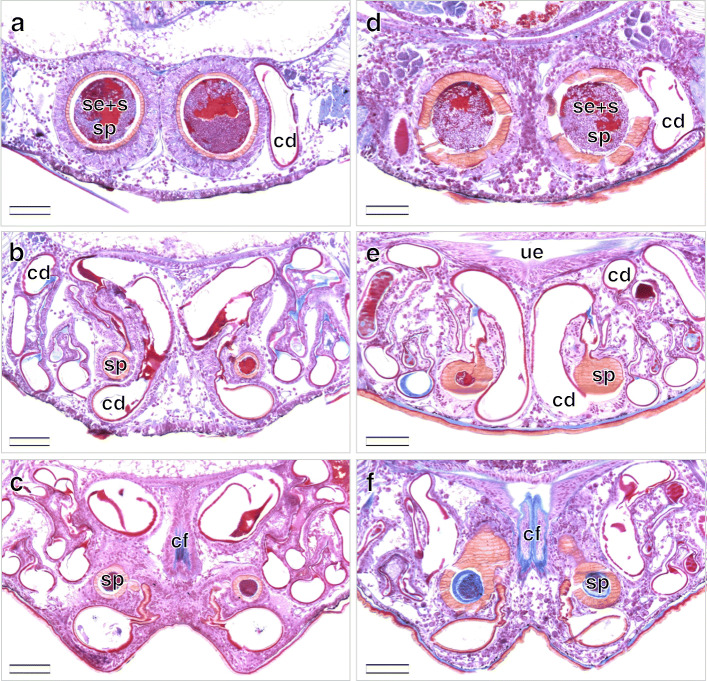
Serial cross sections of the genitalia of mated *L. geometricus* females in different developmental stages starting from the anterior. Dorsal is at the top, ventral is at the bottom. **a**-**c**: Spermathecae of a mated late subadult female are both filled with spermatozoa that are imbedded in a red-pink secretion. Additionally, some areas are stained in red-orange. **d**-**f**: Spermathecae of a mated adult female are both filled with spermatozoa that are imbedded in a red-pink secretion. Additionally, some areas are stained in red-orange. **f**: Posteriorly, the spermathecae are predominantly filled with a bright blue secretion. Scale bars = 100 μm. cd, copulatory duct; cf., common fertilization duct; s, spermatozoa; se, secretion; ue, uterus externus; sp., spermatheca

**Fig. 9:**
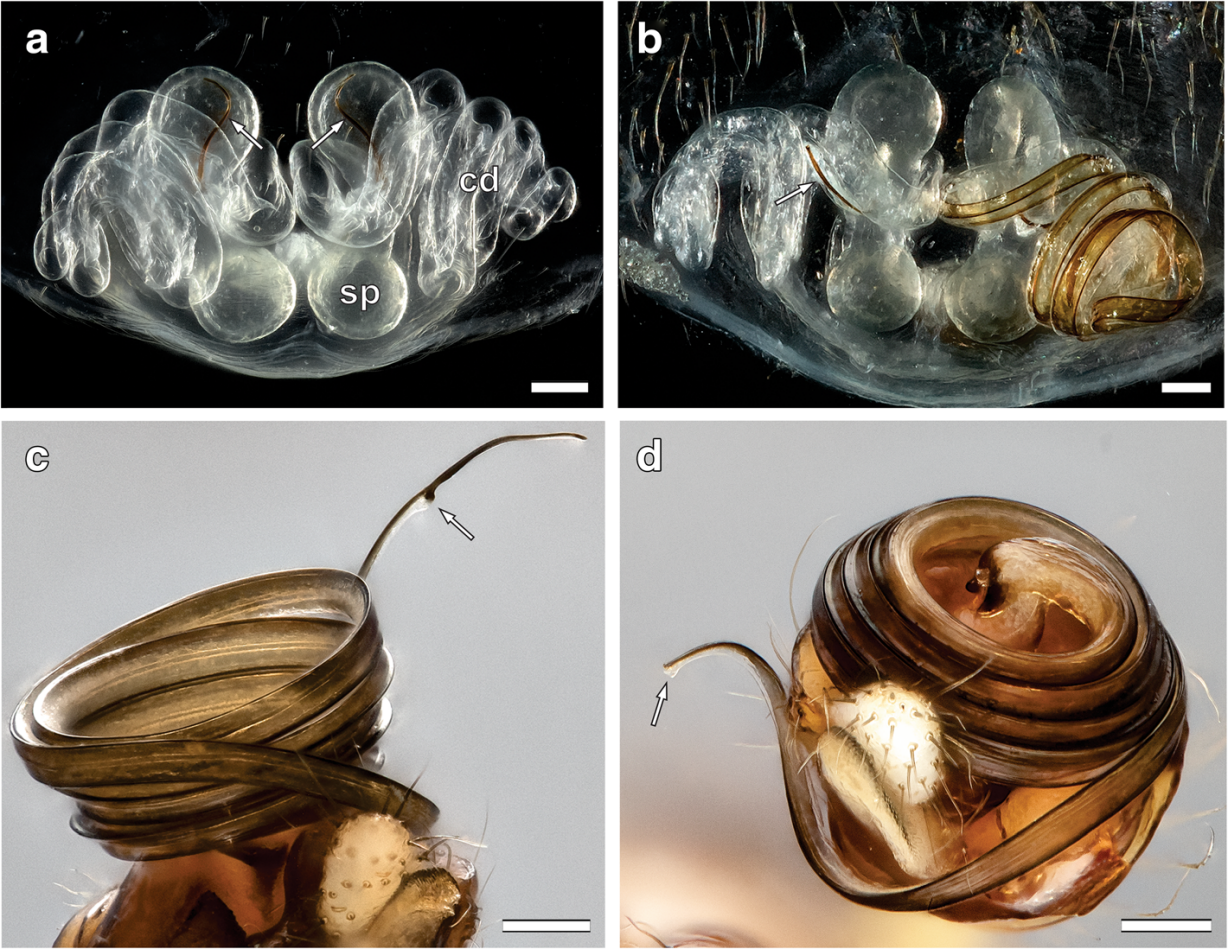
**a**, **b**: Cleared epigynum with paired spermathecae and copulatory ducts of mated *L. geometricus* females, showing different plug types and plug positions. Dorsal view. Anterior is at the top, posterior at the bottom. **a**: Female genitalia containing an embolus tip in each spermatheca (arrows). Emboli are located at the spermathecal entrance and extending to the most anterior inner wall. **b**: Female genitalia containing male plugs that are located inside the copulatory duct, showing an embolus tip on the left side (arrow) and a whole embolus on the right side. **c**, **d**: Different states of the sperm transfer organ (emboli) on the male pedipalp after mating. Ventral view. **c**: Embolus with intact tip. Arrow points to the thickened breaking point. **d**: Embolus missing the tip (arrow). Scale bars = 0.1 mm

Both late-subadult and adult females possess copulatory ducts, spermathecae and fertilization ducts (Fig. 4; but see below for more subtle differences between the two stages). Each copulatory duct spirals from the copulatory opening towards and around a spermatheca and connects to it (Fig. 4). As can be seen from the histological sections, the copulatory duct is not a closed tube but a spiralling fold consisting of a broad, functional duct part and a connecting part (Figs. 5a, c, e, 6b, c, e, f, 7a, d, 8b, c, e, f). The duct part hosts the male embolus during copulation. The connections between the functional ducts are hidden between their coils (Figs. 4, 9a, b). Each spermatheca is dumb-bell-shaped consisting of an anterior and posterior lobe connected by a narrow middle region [21] (Figs. 2, 4, 9a, b). The copulatory duct connects to the anterior lobe of the spermatheca close to the middle region (Fig. 2). Before the posterior lobe, the spermathecal cuticle forms a short, sclerotized fertilization duct (Fig. 4). The fertilization ducts connect to the medially-located common fertilization duct (Fig. 6c, f) which leads to the uterus externus (Fig. 6c). Muscle fibres extend from the common duct to the epigynal plate (Fig. 6c, f).

#### Maturation of female genitalia

In early-subadult females, the cuticle of the copulatory ducts and spermathecae is not yet formed, however, the course of the copulatory ducts and the shape of the spermathecae are already outlined by an invaginated epidermal epithelium (also termed hypodermis) consisting of elongated cells that stain in dark violet with Azan (Fig. 5a, b) (see also [21, 22]). The lumina they enclose are the prospective lumina of the copulatory ducts and spermathecae. A substance staining in light blue is abundant in the spermathecal lumen (Fig. 5b), and also occurs in the lumina of the copulatory ducts, where it appears contracted and more fibrous (Fig. 5a, b).

In late-subadult females, the hypodermal cells show a thin cuticle towards the lumina of both ducts and spermathecae. The cuticle stains bright red along the ducts and orange along the spermathecae (Fig. 5c, d), suggesting different degrees of sclerotization. The spermathecal lumen is filled with a light-blue substance (Fig. 5c, d) with occasional red droplets.

In adult females, the epidermis surrounding the spermathecae appears less prismatic, more vacuolised and stains paler violet than in both subadult stages (Fig. 5b, d versus 5F). Compared to the epidermis of the copulatory duct, the narrow connection to the spermathecae is highly sclerotized and shows an epithelium of densely arranged and elongated cells (Fig. 6e) similar to late-subadult females (Fig. 6b). The cuticle of the spermatheca is much thicker in adult females compared to late-subadult females and exhibits numerous pores (Figs. 5f, 6d). The pores host epithelial cells that seems to discharge a red staining substance into the bright blue secretion of the spermathecal lumen (Figs. 5f, 6d). Bright blue secretion also occurs in smaller amounts in parts of copulatory ducts (Fig. 6e, f).

#### Mated females

After mating, in all late-subadult (*N* = 5) and adult females (*N* = 8), at least one spermatheca contained spermatozoa. Typically, the anterior lobe - to which the copulatory duct connects (Fig. 2) - and the middle region of a spermatheca contained large quantities of sperm, while the amount of sperm in the posterior lobe - to which the fertilization duct connects (Fig. 4) - varied considerably (see below). In spermathecae that did not contain sperm or only a small amount of sperm in the anterior lobe, spermatozoa were found neither in the middle region nor in the posterior lobe of the spermathecae.

Large amounts of sperm were found embedded in a red-orange substance in both late-subadult and adult females after mating (Figs. 7b, e, 8a, d). In five adult females (*N* = 8) a small amount of blue substance was also present in the anterior lobe of one or both spermathecae. In four late-subadult (*N* = 5, 80%) and five adult females (*N* = 8, 62.5%) both spermathecae contained sperm. In one of the adult females, however, the anterior lobe of one spermatheca contained only a few spermatozoa embedded in blue substance with a few red droplets. When no sperm was found, a blue substance with few red droplets was present, as typical for virgin females.

A tip of the embolus was found in two late-subadult (*N* = 5) and three adult females (*N* = 8) at the entrances to the anterior lobes of both spermathecae, both of which contained sperm (Fig. 7c, f). Whenever embolus tips were present in spermathecae, they always occurred together with sperm but embolus tops did not always occur when sperm was present. The embolus tips were always located in the narrow connection between the copulatory duct and the spermatheca, and extended into the lumen of the anterior lobe (Figs. 7c, f, 9a).

If sperm was present in the anterior lobe, the middle narrow region of the spermathecae also contained spermatozoa as well as small amounts of red secretion (Fig. 8b, d). These materials either filled the entire region or were surrounded by blue substance. In one spermatheca of one adult female, however, the narrow region contained predominantly bright blue substance with few red droplets and no sperm, while in another adult female the narrow region of one spermatheca contained sperm within bright blue substance.

Irrespective of the developmental stage at mating, the posterior lobe of the spermathecae of mated females was mostly partly or entirely filled with spermatozoa and associated red substance as well as blue substance (Fig. 8f). In three late-subadult females (*N* = 5, 60%) sperm was present in large amount in the posterior lobe of one (*N* = 1) or both (*N* = 2) spermathecae. In four adult females (*N* = 8, 50%) the sperm in the posterior lobe was present in one (*N* = 2) or both (*N* = 2) spermathecae.

### Spermathecae

#### Spermathecal area

The areas of the anterior lobe and the posterior lobe of the spermathecae did not differ between late-subadult (*N* = 11) and adult females (*N* = 14) (Wilcoxon signed-ranked test; anterior lobe, W = 46.00, *P* = 0.10; Fig. S1A; posterior lobe, W = 61.00, *P* = 0.40; Fig. S1B). There was also no significant difference between females of different developmental stage and mating status in the areas of anterior or posterior lobes (Kruskal Wallis test; anterior lobe: χ^2^ = 3.21, *P* = 0.36, Fig. S1A; posterior lobe: χ^2^ = 2.31, *P* = 0.51, Fig. S1B).

#### Spermathecal cuticle thickness

**Fig. 10:**
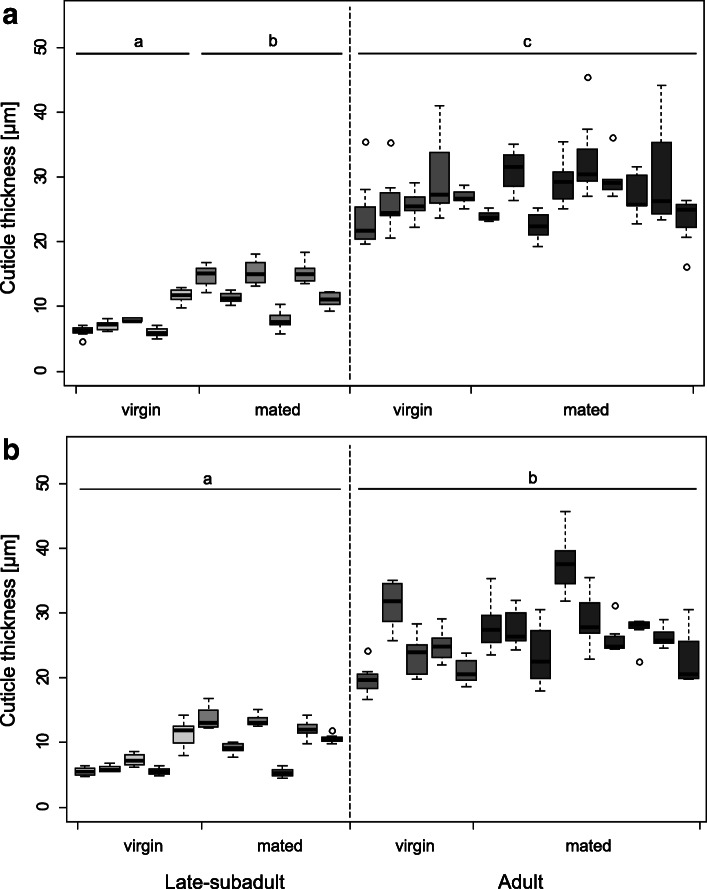
Cuticle thickness of the anterior (**a**) and posterior lobe (**b**) of the right spermatheca as a function of the female’s developmental stage and mating status. Each boxplot represents repeated measures around one spermatheca of an individual. Different letters indicate statistically significant differences

The spermathecal cuticle was significantly thinner in late-subadult females (*N* = 11) than in adult females (*N* = 14) both in the anterior (ANOVA: F = 156.00, *P* < 0.01; Fig. 10a) and posterior lobe (ANOVA: F = 112.98, *P* < 0.01; Fig. 10b). Overall, the cuticle thickness differed among the females of different developmental stages and mating statuses (ANOVA: anterior lobe F = 67.76, *P* < 0.01; posterior lobe ANOVA: F = 44.79, *P* < 0.01). As to the anterior lobe, the cuticle was thinner in virgin late-subadult females, intermediately thick in mated late-subadult females and the thickest in adult females (Table S1; Fig. 10). As to posterior lobes, the cuticle was thinner in late-subadult females compared to adult females with no difference between mating status in both developmental stages (Table S1; Fig. 10).

#### Spermathecal epithelium thickness

**Fig. 11:**
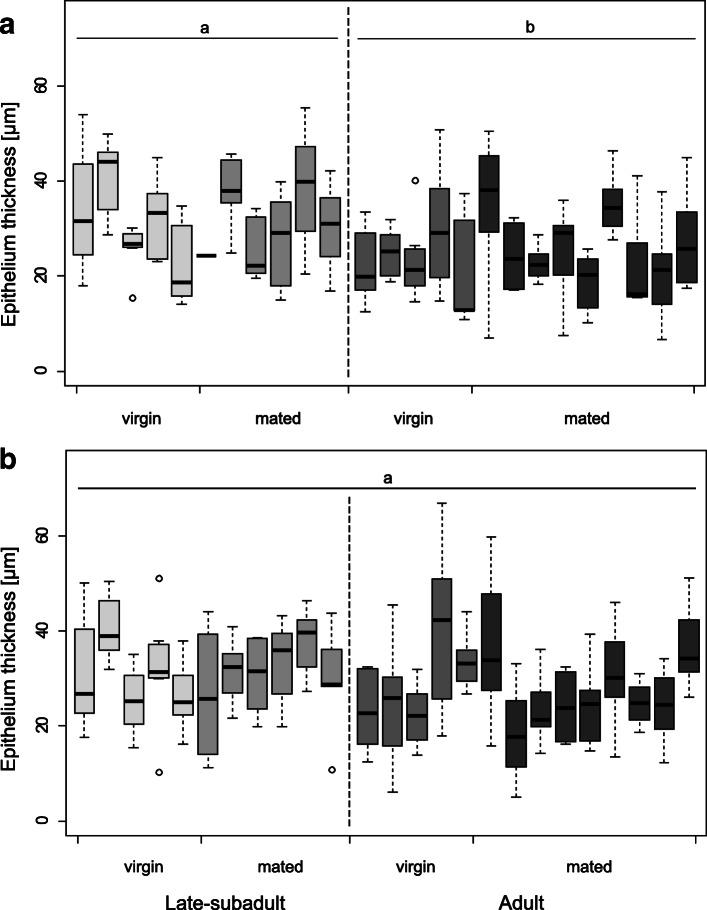
Epithelium thickness of the anterior (**a**) and posterior lobe (**b**) of the right spermatheca as a function of the female’s developmental stage and mating status. Each boxplot represents repeated measures of a single individual. Different letters indicate statistically significant differences

In comparison to adult females, the epithelium of late-subadult females was significantly thicker in the anterior (ANOVA: F = 6.98, *P* = 0.01; Fig. 11a) but not in the posterior lobe (ANOVA: F = 2.31, *P* = 0.14; Fig. 11b). When separating the females into virgin and mated females of the two developmental stages there was no significant difference in epithelium thickness, neither in the anterior (ANOVA: F = 2.19, *P* = 0.12; Fig. 11a) nor in the posterior lobe (ANOVA: F = 0.79, *P* = 0.52, Fig. 11b).

### Mating plugs

Successful matings occurred in 53 of 60 (88.3%) trials with late-subadult and 37 of 42 (88.1%) trials with adult females, which was not significantly different (Chi^2^ test: χ^2^_(1)_ < 0.01, *P* = 0.97).

#### Embolus breakage in males

**Fig. 12:**
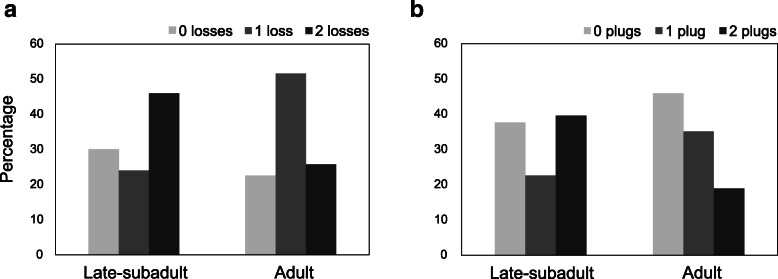
Percentage of (**a**) male’s embolus losses (*N* = 81) and (**b**) plugs found in female genitalia (*N* = 90) as a function of the female’s developmental stage

The probability of breaking off the tip or the whole embolus during mating did not differ significantly between males that mated with late-subadult females (35 of 50; 70%) and males that mated with adult females (24 of 31; 77.4%) (Chi^2^ test: χ^2^_(1)_ = 0.53, *P* = 0.47). Mostly, males were missing only the tip of the embolus (Fig. 9a, d). In 14.3% (5 of 35) of males mated to late-subadult females and 29.2% (7 of 24) of males mated to adult females, a larger piece or the entire embolus was missing (Fig. 9b). The proportion of a small or large part of the embolus breaking off did not differ significantly between males mated to late-subadult and adult females (Chi^2^ test: χ^2^_(1)_ = 1.95, *N* = 59, *P* = 0.16). However, significantly more males lost both emboli (tips or larger parts) when mating with late-subadult females (23 of 35; 65.7%), compared to males mating with adult females (8 of 24; 33.3%) (Chi^2^ test: χ^2^_(1)_ = 5.99, *P* = 0.01, Fig. 12a).

Males that mated with late-subadult females were similarly likely to deposit the broken off embolus in the genitalia of the female. Plugging success was 85.7% (30 of 35) in late-subadult matings compared to 66.7% in matings with adult females (16 of 24; Chi^2^ test: χ^2^_(1)_ = 3.01, *P* = 0.08).

#### Plugs in the female genitalia

Overall, one or two embolus pieces were found in the macerated genitalia of 53 out of 90 (58.8%) females. In 92.5% of cases (49 of 53), the parts consisted of a tip of the embolus and in 7.5% of cases of a larger part of the embolus (4 of 53). Plugs occurred in 62.3% of late-subadult females (33 of 53) and 54.1% of adult females (20 of 37); the occurrence of mating plugs did not significantly differ between the developmental stages of the female (Chi^2^ test: χ^2^_(1)_ = 0.61, *P* = 0.44; Fig. 12b). The plugs were located at the entrance to the spermatheca in 93.9% of subadult females (31 of 33) and in 95% of adult females (19 of 20), and in the remaining cases inside the copulatory duct. There was no significant difference in plug position between the groups (Fisher’s exact test: *P* = 1.00). Females that mated in the subadult stage had both spermathecae plugged with significantly higher probability (21 of 33; 63.6%) than adult females (7 of 20; 35.0%) (Chi^2^ test: χ^2^_(1)_ = 4.10, *N* = 53, *P* = 0.04; Fig. 12b).

## Discussion

Internal genitalia of female spiders develop in the course of the subadult stage [21, this study], and are considered non-functional until after the final moult [23]. In the brown widow spider, *L. geometricus*, early-subadult females lack the cuticular inner genital structures of adult female genitalia; however, in the late-subadult stage all components of the inner genital system (copulatory ducts, spermathecae and fertilization ducts) are already developed below the cuticle. During the moult to adult stage the cuticle is shed but the underlying genital system is not affected by the moult. Consequently, females that mated during the late-subadult stage retain sperm even after their final moult. This is corroborated by the behavioural observation that they are able to copulate and produce viable offspring similar to adult females [10]*.* Nevertheless, our histological comparison of the genitalia of late-subadult and adult females revealed significant differences in the structural equipment of their spermathecae. The thickness of the cuticular spermathecal wall and the appearance of secretions within the spermathecae differ between the developmental stages. These differences reflect ongoing maturation of the genitalia in late-subadult females and might result in differences in sperm storage conditions between the late-subadult and adult females. Furthermore, males inseminated and plugged both spermathecae with significantly higher probability in matings with late-subadult females than in matings with adult females.

Several authors described external and internal genitalia in adult widow spider females, showing that the gross morphology is very similar across the species of this genus (e.g. [21, 22, 24]). As in other congeners, in adult females of *L. geometricus* the epigyne (external genitalia) is composed of a heavily sclerotized protuberance and a cave-like atrium with two copulatory openings*.* In early-subadult females, this area is pale and rather flat, but gradually becomes elevated and already up to six days before the moult to adulthood (i.e. in the late-subadult stage) the elevated part darkens indicating that the epigyne beneath the cuticle is formed and sclerotized. Immature mating is observed only during the late-subadult stage [10, 13]. The epigyne, however, is covered by the exoskeleton at this stage, hence there is no direct access to it. Males bite through the cuticle covering this area and thereby expose the atrium with the two copulatory openings, into which they insert one of their copulatory organs (video S1). Similar behaviour was observed in *L. hasselti* [10] and *L. hesperus* (Baruffaldi, pers. communication), suggesting that males of these widow spiders use their chelicerae to get access and enable insertion of the pedipalps into the concealed genital openings of late-subadult females. Once the cuticle is opened, the male can insert his embolus in the same way as during matings with adult females, since the exposed copulatory openings lead via fully formed copulatory ducts to the spermathecae. Late-subadult females are functionally ready for copulation and no alternative route (e.g., through piercing female body cavity [15]) is employed. Indeed, in mated subadult females we found no injuries other than the ripped open cuticle just above the atrium.

Although able to mate, late-subadult females still have to undergo one more moult to reach the adult stage. During moulting, not only the body cuticle is shed but also all interior cuticular lining of structures such as booklungs, sensilla and ectodermal parts of the gut (e.g., [20, 25, 26]). Spermathecae are also lined with cuticle and, in some basally branching off lineages of spiders, this cuticle is shed with the rest of the exoskeleton when females continue to moult after reaching maturity (e.g. [19, 20]). Post-maturity moulting has been occasionally observed in araneomorph spiders (including *Latrodectus*) [26], but mostly was considered an anomaly. However, it regularly occurs in the giant wood spider *Nephila pilipes*, in which the sclerotized part of the genitalia is not shed during the post-maturity moult. Consequently, *N. pilipes* females retain previously gained sperm despite the extra moult [27, 28]. Although this case concerns adult females and post-maturity moulting, it suggests that, even if the genitalia are formed already in the late-subadult stage, the spermathecae are not necessarily moulted and their contents can be retained. The shed cuticle of the post-maturity moulting females of *N. pilipes* bears no ducts or spermathecae, instead, there is a hole in the genital area [27]. Likewise, the moulted cuticle of the subadult stage of *L. geometricus* bears no signs of genital structures.

Internal genitalia of subadult *Latrodectus* females were previously inspected in *L. curacaviensis* showing that the cuticular spermathecal walls are secreted by invaginated epithelial cells already during the subadult stage, but fully developed, sclerotized genitalia were reported only from adult females [21]. Our investigation of early-subadult female genitalia in *L. geometricus* revealed that cuticular structures are not present at this point but the shape and position of the copulatory ducts and spermathecae are already outlined by the epithelium. In late-subadult females, the cuticular aspects of ducts and spermathecae are very similar to the adult stage (but see below). Apart from copulatory ducts and spermathecae, fertilization ducts and associated muscles also appear to be fully formed in late-subadult females (not shown).

While the copulatory ducts in *L. geometricus* are lined by a similarly thin cuticle in late-subadult and adult females, the spermathecal walls are several times thicker in adult females. The cuticle is secreted by the hypodermal epithelium around the spermathecae [21]. Moreover, in spiders and many other arthropods this epithelium is typically equipped with glandular tissue that discharges secretion via pores through the cuticle into the spermathecal lumen (e.g. [21, 29–33]). We found no significant differences in the thickness of the glandular tissue around the spermathecae of late-subadult and adult females. However, the glandular cells in late-subadult females appear less vacuolised than in the adult females. This may indicate, that the glandular cells of late-subadult females produce less or different secretions than glandular cells of adult females. Indeed, the Azan staining resulted in light blue staining of late-subadult virgin spermathecal content, while the content of adult virgin spermathecae stained bright blue, indicating that both secretions are acidophilic [34] but differ in composition.

The function of the secretions in spider spermathecae is largely unknown, but as in insects (e.g. [35]) they are considered crucial for successful fertilization, serving to maintain, activate and/or transport stored sperm [30, 32, 36–38]. Secretions are reported to be building up in female pholcid spiders only after the final moult, which coincides with the females’ readiness to mate [39]. However, in *L. geometricus*, secretions are already apparent days before the final moult and it is as yet unclear if this is the exception in spiders. Since late-subadult females of *L. geometricus* differed in spermathecal content compared to adult females it is likely that males transfer their sperm into different environments when mating with females of different stages. The storage conditions are obviously sufficient for successful fertilization later on, since immaturely-mated females produced offspring in numbers similar to [10, 11, 14] or even higher than [12] adult-mated females after a single mating. Since widow spider females are often polyandrous [7], it needs to be tested if ejaculate stored in immature genitalia is disadvantaged in sperm competition, if the female re-mates with another male after the final moult.

We expected insemination failures, for example, sperm that was transferred but did not reach the spermathecae (e.g. [40]), to be more common in subadult-mated females due to ongoing maturation and/or differences in mating behaviour. When mating with immature females, males do not somersault [10] and the absence of this behaviour may mechanically alter the insertion pattern (e.g. the depth of the insertion) and so affect the location where sperm is stored and also the placement of mating plugs within the female reproductive tract. However, in mated females of both stages we found sperm only in the spermathecae, never in the copulatory ducts, suggesting that males rarely fail to inseminate female spermathecae.

The distribution of sperm within the spermathecae appeared similar in both stages and the secretions present in spermathecae containing sperm stained similarly with Azan staining. We did not count the spermatozoa, but the gross amount of sperm transferred seemed to be higher in the late-subadult females., since 80% of late-subadult and 63% adult females showed sperm in both spermathecae. The apparent overall higher number of sperm in late-subadult spermathecae might result from the fact that males mating with these females are not interrupted by female cannibalism [10] and achieve a higher number of insertions on average.

In experiments with adult females of *L. hasselti* mated to two males, first male sperm priority was detected and the first male fathered on average 80% of the offspring [41]. This sperm precedence pattern likely arises from plugging of the spermathecae that impedes sperm transfer in second males [41]. Since immature female genitalia are still developing, we expected mating plugs to be less common than in matings with adult females, for example due to less sclerotized genitalia in which the embolus tips cannot break off or be placed effectively. We observed that some males lacked embolus tips after mating but the tips were not found within the genitalia of females they mated with. However, the loss of an embolus tip and its absence in female genitalia did not differ between males that mated with adult or subadult females. Larger parts of the emboli were rarely (14.8%) broken off and this breakage did not differ between female developmental stages. A third of these larger embolus parts was found in the female genitalia where they likely represent an effective barrier. However, this loss prevents the male from copulating again with that palp if he survives copulation. The loss of the embolus tip, on the other hand, does not seem to preclude insemination [7] and allows a male to father additional offspring if he survives the copulation.

We hypothesized that plugs in the late-subadult genitalia may be displaced or lost during the moult to adult stage. This is not likely since the genitalia are not moulted with the rest of the cuticle. In both stages, the plugs were mostly placed at the spermathecal entrance where they are highly effective. Only rarely they were found in copulatory ducts or inside the spermathecae, where they cannot prevent insertion by a second male. In addition, plugging occurred with similar frequency in both stages: 62% of late-subadult and 54% of adult once-mated females had at least one plug. However, in late-subadult females typically both spermathecae were plugged (64%), while this was the case in only 35% of adult females. This difference does not result from differing morphology of the two stages, but rather from different mating behaviour. In adult females, males trigger cannibalism by somersaulting, which limits the males to a single insertion but also reduces the females’ willingness to re-mate [4]. Males do not sacrifice themselves to late-subadult females thus these females may remain receptive to other males. However, in immature mating males often perform two insertions, one with each pedipalp [10], which allows them to inseminate and plug both spermathecae and so re-mating might be severely hampered. However, since we investigated once-mated females, it remains unclear what happens to the plug if another male attempts to insert his embolus and whether the effectiveness of the deposited plugs is the same in both female developmental stages. If a subsequent male nevertheless succeeds in inseminating plugged spermathecae, the amount of sperm might be relatively small compared to the amount transferred by the first male that was not interrupted by cannibalism. This potential numerical advantage in sperm competition, combined with the first-male sperm precedence observed in the congener *L. hasselti* and with the higher plugging success, might have selected for the loss of male self-sacrifice behaviour when mating with late-subadult females.

## Conclusions

Although differing in some respects from adults, the genitalia of subadult females of *L. geometricus* are functionally ready to mate several days before the moult to adult stage. Considering that the morphology of internal and external genitalia is very similar across widow spider species*,* immature mating is likely widespread within the genus *Latrodectus*.

We found differences in the secretions inside the spermathecae of late-subadult and adult females, which may reflect different environmental conditions for the transferred sperm. Sperm storage conditions may lead to differential competitive advantage under sperm competition, provided that the female mates with another male before or after the final moult. However, males mating with late-subadult females are more likely to plug both female spermathecae, leading to a higher potential of monopolizing the sperm storage sites and thereby securing paternity. To answer these questions, our future study will focus on paternity shares in double-mated females differing in the timing of their first and second mating.

## Methods

Females and egg sacs of *Latrodectus geometricus* C. L. Koch, 1841 were collected in central Israel (Bat Yam, Be’er Yaakov, Rishon Le’Zion, Ramat Gan and Ma’agan Michael) and brought to the laboratory at the Sede Boqer Campus of Ben-Gurion University of the Negev, Israel. They were housed individually in plastic boxes in a climate chamber (25 ± 1 °C, 65% relative humidity and 14:10 h light:dark) and were fed twice a week with flies or grasshopper nymphs. After oviposition and hatching of spiderlings from the egg sacs, young spiders were kept together for the first two instars. We transferred spiderlings to individual plastic containers after they had reached the third instar. The spiders were transported to the University of Greifswald, Germany, and maintained in a climate chamber at 25 ± 1 °C under reversed 12:12 h light:dark and 60% relative humidity. Their webs were sprayed with water twice a week. We recorded the date of the moult to the subadult stage and the final moult of each spider. Spiderlings and males were fed twice a week with six fruit flies (*Drosophila hydei*). Subadult females were fed with two *Lucilia sp.* flies, mature females with one *Protophormia* fly or one small sized cricket *Achaeta domestica* (7–10 mm) twice a week.

Female external genitalia are situated on the ventral side of the opisthosoma [23]. As early-subadult females, we refer to females max. seven days after their moult to the subadult stage when their genital area is pale grey. Late-subadults are females min. ten days after the moult to the subadult stage, which is approximately four days before the moult to adulthood when immature mating may occur (see [10] for details). The genital area of late-subadult females is dark brown. Adult females moulted to the adult stage and showed the species-specific external genital plate, the epigyne, with copulatory duct openings. Adult females were used in the experiments no earlier than seven days after their final moult.

To obtain mated individuals, we paired virgin late-subadult and adult females with virgin males. Five days prior to the mating trials, females were transferred to clean experimental boxes (10 cm × 10 cm × 6 cm). The females were fed one day before they were transferred into the experimental boxes and no food was provided while in the boxes. Since *L. geometricus* are nocturnal [42], males were introduced into the box at the beginning of the dark phase and they were left there for 24 h. Afterwards, late-subadult females were inspected for disrupted cuticle on their genital region and adult females for the occurrence of cannibalism of the male. Both features indicate that mating had occurred. These females were anaesthetised with carbon dioxide and processed further (see below).

### External female genitalia

External genitalia of one virgin early-subadult female, one virgin and one mated late-subadult female and one virgin and one mated adult female were fixed in 70% ethanol and photographed using a customised Visionary Digital BK Plus imaging system (Dun, Inc., Palmyra, Virginia, USA). All image adjustments were carried out using either Adobe Photoshop CS6 and Illustrator CS4 (Adobe systems, Inc., San José, California, USA) or CorelDRAW 2017 and Corel PHOTO-PAINT 2017 (all Corel Corp., Ottawa, Ontario, Canada). Image stacking was processed using “Zerene Stacker” stacking software (Zerene Systems LLC, Richland, Washington, USA).

We inspected cuticles shed by virgin (*N* = 15) and mated (*N* = 15) subadult females during their final moult for a presence of spermathecae under a stereomicroscope (Carl Zeiss stereomicroscope 475,110–9904).

### Internal female genitalia

#### Paraffin histology

Opisthosomata of five virgin late-subadult, five virgin adult, five mated late-subadult and eight mated adult females were separated from the prosoma and the cuticle was punctured several times to allow penetration of the fixative Duboscq-Brasil (picric acid, 80% ethanol, 40% formol and acetic acid) [43]. After a minimum of one week in the fixative, the samples were dehydrated and washed in a graded series of ethanol concentrations as follows: 80% ethanol for 2 h, 96% ethanol for 30 min, 96% ethanol for 30 min, 96% ethanol and ≥ 99.5% tetrahydrofuran for 2 h, tetrahydrofuran for 18 h. The samples were then transferred into 1:1 solution of heated paraffin and tetrahydrofuran and placed in a heating cabinet (63 °C) for 24 h. Finally, the solution was exchanged with heated paraffin and stored in a heating cabinet (63 °C) for at least 1 day [34, 44]. The sample was then placed into an embedding mold filled with paraffin where it was left to harden for 2 days before sectioning. The block was trimmed and transversal sections were produced with a microtome (Microm HM 360) filled with distilled water heated up to 40 °C. Sections were 5 μm thick and cutting speed was 30 mm/s. The section ribbons were placed on object slides coated thinly with protein-glycerin to increase the adherence of sections. The object slides were then transferred onto a heating plate (40 °C) for at least 20 min.

To remove the paraffin, the object slides were immersed into Roti-Histol for 10 min and were transferred for 5 min into 2-Propanol, 96, 80 and 60% ethanol and finally into distilled water. For staining, the slides were transferred into nuclear fast red-aluminium sulphate for 30 min, which stains the cell nucleus red [45]. After washing with distilled water, a 10 min treatment with phosphotungstic acid (5%) followed to bleach and stain connective tissue. The sections were washed with distilled water and treated with aniline blue-orange G-acetic acid for 10 min, which stains e.g. cytoplasm and connective tissue [45, 46]. The sections were then immersed in distilled water and dehydrated in 60, 80, 96% ethanol and 2-Propanol, each for 5 min. The sections were transferred into Roti-Histol for 5 min, then coated with Roti-Histokitt II and protected by cover slips. Selected paraffin sections were photographed using a customised Visionary Digital BK Plus imaging system and the images were adjusted as described above.

In the genus *Latrodectus*, a spermatheca has a dumb-bell shape consisting of an anterior (AL) and posterior lobe (PL) connected by a narrow middle region [21]. Consequently, for females of different developmental stages (late-subadult, adult) and mating status (virgin, mated), we measured the area of the lumen, the thickness of the cuticle and the surrounding epithelium for each spermathecal lobe separately. For the measurements, we chose the mid histological section of each lobe of the right spermatheca. The sections were photographed with a light microscope (Olympus BX60 Fluorescence Microscope, software AxioVision 4.8). To assess the spermathecal lobe area, we used the contour line tool (AxioVision 4.8), tracing along the inner side of the spermathecal cuticle. To receive an estimate of the thickness of the cuticle and the surrounding epithelium, we defined a central point within each spermathecal lobe area as the crossing point of two perpendicular axes of maximum length. To assess the thickness of cuticle and epithelium, we attempted to take eight measurements around each lobe (at 45 degrees) with each measuring axes starting from the central point of the spermathecal lobe. Since some spermathecal lobes were partly ruptured from sectioning, the data are based on less than eight measurements per spermatheca on average (mean + SD for cuticle AL: 7.48 ± 1.16; cuticle PL: 7.52 ± 0.71; epithelium AL: 7.36 ± 1.58; epithelium PL: 7.24 ± 1.45) for both late-subadult and adult females.

#### Statistical analyses

To test the effect of female developmental stage on the spermathecal area, cuticle and epithelium thickness of the anterior and posterior spermathecal lobe, we pooled virgin and mated females together within a given stage (virgin and mated late-subadult versus virgin and mated adult females). Further, we tested the effect of female developmental stage in combination with the mating status (virgin late-subadult, virgin adult, mated late-subadult and mated adult) on the measured variables.

The effect of the female developmental stage on the area of anterior and posterior lobe was tested with a Wilcoxon signed-rank test. The effect of the developmental stage and mating status was tested with a Kruskal Wallis test. To test the effect of the female developmental stage only and female developmental stage in combination with mating status on cuticle and epithelium thickness of the anterior and posterior spermathecal lobe, linear mixed-effects models (LMMs) were used in R (R Core Team, 2019, version 3.6.1, software RStudio). The models were created using the R package lme4 [47]. To account for repeated measures of the same individual, female ID was included as a random effect. Two-way ANOVA (Type III analysis of variance table with Satterthwaite’s method) based on the LMM was used to test for statistical significance. The *p*-values of ANOVA were obtained by means of the R package lmerTest [48]. The normal distribution of the data was assessed by means of QQ-plots. If the model showed a significant difference between the groups, Tukey post hoc tests were applied, using the R package multcomp [49] to identify differences between the females of different developmental stage and mating status.

#### Micro-computed tomography

To illustrate and compare the spermathecal morphology between the female developmental stages in situ, opisthosomata of one early-subadult female, five late-subadult females and four adult females were fixed in Duboscq-Brasil [43] and kept in 80% ethanol. After dehydration, samples were contrasted overnight using a 1% iodine solution (in pure ethanol). After washing in 80, 90 and 100% ethanol, the samples were critical point dried with a BAL-TEC CPD 030 and mounted on insect pins using super glue. Scans were performed in an Xradia XCT-200 (Carl Zeiss Microscopy GmbH) using the 4x and 10x objective lens unit with the following scan parameters: 40 kV, 8 W, 200 μA, exposure time 7 s/frame. Reconstructed image stacks were created using XMReconstructor software (Carl Zeiss Microscopy GmbH). Data were visualized and processed using the 3D analysis software AMIRA 5.6 (Visualization Science Group, FEI).

### Mating plugs

To analyse the frequency of embolus breakage and plugging in *L. geometricus* depending on the developmental stage of the female, we staged matings with late-subadult (*N* = 60) and adult females (*N* = 42) using virgin males (*N* = 102). After mating, males and females were fixed in 70% ethanol. The copulatory organs of 50 males mated to late-subadult females and 31 males mated to adult females were checked under a stereomicroscope (Carl Zeiss) to establish the occurrence of embolus loss. It was recorded whether a pedipalp lacked the tip or a larger part of the embolus. In all mated females (53 late-subadult and 37 adult), the genital area with the underlying spermathecae and copulatory ducts were dissected out with a micro-scissors and transferred into a vial containing 5% potassium hydroxide. Herein, the genitalia were macerated at 60 °C on a heating plate for two days. The cleared and transparent copulatory ducts and spermathecae were transferred into 70% ethanol and inspected for mating plugs under a stereomicroscope (Carl Zeiss stereomicroscope 475,110–9904). We recorded the occurrence, number and size of embolus pieces, as well as their position (spermathecal entrance, copulatory duct). For illustration, selected pedipalps and female genitalia were photographed using a customised Visionary Digital BK Plus imaging system as described above.

#### Statistical analyses

We compared frequencies of loss of the embolus or its tip, and loss of one versus both emboli (or their tips) between males mating with late-subadult and adult females. Further, we compared the occurrence of emboli ending up in the female genitalia (no plug versus one or two plugs, be they tips or the whole embolus), the number of plugs (one plug versus two plugs) and the plug position (spermathecal entrance versus copulatory duct) between late-subadult and adult females. The plugging success was assessed by comparing the number of embolus losses in a male and the occurrence of the embolic structures in the respective females. If all embolic structures that were missing in the male were found in the female this was categorized as plugging success. All comparisons were tested with Chi^2^ tests or Fisher’s exact tests in SPSS.

## Supplementary Information

**Additional file 1: Figure S1**. Area of the anterior (A) and posterior lobe (B) of the right spermatheca as a function of the female’s developmental stage and mating status. Each dot represents a single measurement of an individual female.

**Additional file 2: Table S1**. Differences between the females of different developmental stages (late-subadult, adult) and mating status (virgin, mated) based on Tukey post hoc tests.

**Additional file 4: Dataset S1**. Data generated and analysed during the current study.

## Data Availability

The datasets supporting the conclusions of this article are included as a supplementary file.
