# Supplementary figures and images for: Functional morphology of immature mating in a widow spider
Source: Front Zool. 2021 Apr 26;18:19. doi: 10.1186/s12983-021-00404-1 (PMC8074507; doi:10.1186/s12983-021-00404-1)

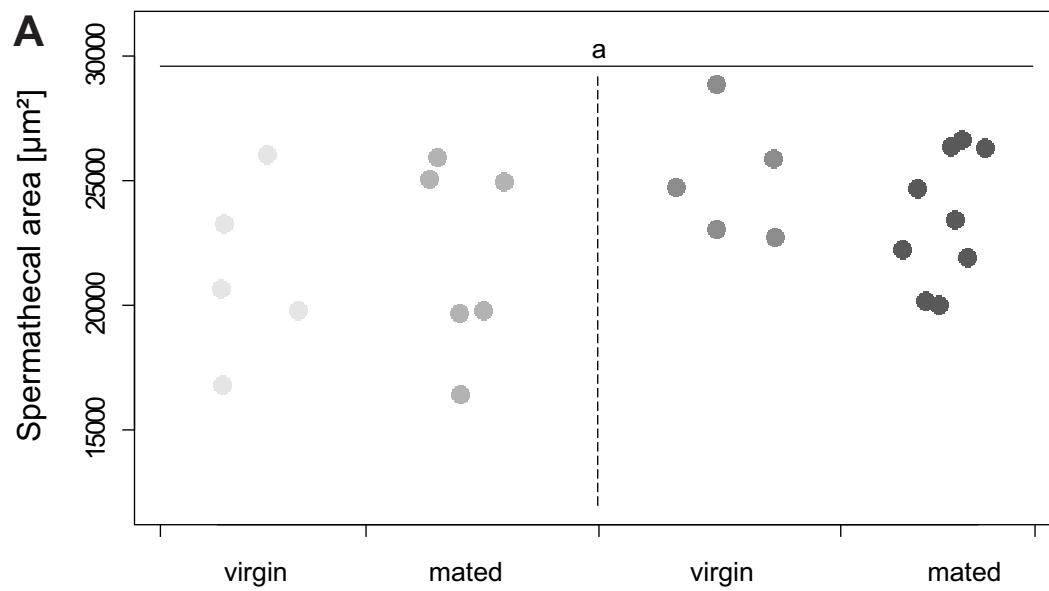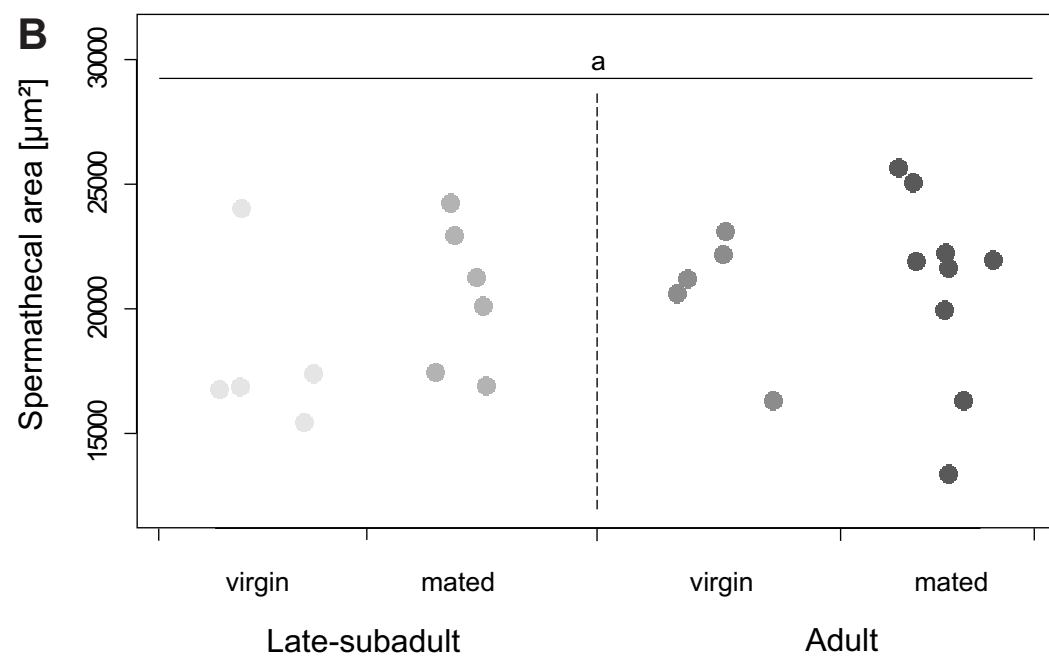

Supplement: Supplementary file 2 — Additional file 1: Figure S1. Area of the anterior (A) and posterior lobe (B) of the right spermatheca as a function of the female’s developmental stage and mating status. Each dot represents a single measurement of an individual female. [file 12983_2021_404_MOESM1_ESM.pdf]
