# Supplementary material for: Functional morphology of immature mating in a widow spider
Source: Front Zool. 2021 Apr 26;18:19. doi: 10.1186/s12983-021-00404-1 (PMC8074507; doi:10.1186/s12983-021-00404-1)
Supplement: Supplementary file 3 — Additional file 2: Table S1. Differences between the females of different developmental stages (late-subadult, adult) and mating status (virgin, mated) based on Tukey post hoc tests. [file 12983_2021_404_MOESM2_ESM.docx]

Table S1. Differences between the females of different developmental stage (late-subadult, adult) and mating status (virgin, mated) based on Tukey post hoc tests.

|  | **Cuticle thickness [µm]** | | | |
| --- | --- | --- | --- | --- |
|  | **Anterior lobe** | | **Posterior lobe** | |
|  | **z value** | **P value** | **z value** | **P value** |
| **Subadult virgin vs. subadult mated** | 2.652 | **0.039** | 1.563 | 0.398 |
| **Subadult virgin vs. adult virgin** | 9.839 | **<0.001** | 7.098 | **<0.001** |
| **Subadult virgin vs. adult mated** | 11.955 | **<0.001** | 9.731 | **<0.001** |
| **Subadult mated vs. adult virgin** | 7.639 | **<0.001** | 5.858 | **<0.001** |
| **Subadult mated vs. adult mated** | 9.635 | **<0.001** | 8.513 | **<0.001** |
| **Adult virgin vs. adult mated** | 0.843 | 0.833 | 1.682 | 0.332 |
